# The Biodegradation of Indigo Carmine by *Bacillus safensis* HL3 Spore and Toxicity Analysis of the Degradation Products

**DOI:** 10.3390/molecules27238539

**Published:** 2022-12-04

**Authors:** Chunlei Wang, Sijia Wang, Jieru Zhang, Shumin Jiang, Daizong Cui, Haiqiong Sun, Chengwei Liu, Lili Li, Min Zhao

**Affiliations:** 1 College of Life Sciences, Northeast Forestry University, Harbin 150040, China; 2 Key Laboratory for Enzyme and Enzyme-Like Material Engineering of Heilongjiang, Harbin 150040, China; 3 Lanzhou Lansheng Plasma-Derived Biotherapies Co., Ltd., Lanzhou 730000, China; 4 Institute of Forestry Science of Heilongjiang Province, Harbin 150040, China

**Keywords:** decolorization, spore laccase, tyrosinase, lignin peroxidase, toxicity test

## Abstract

The aims of this article were to investigate *Bacillus safensis* HL3 spore for its capacity to degrade and detoxify indigo carmine and to provide an effective biological agent for the treatment of isatin dye wastewater. *Bacillus safensis* HL3 spore was found to decolorize indigo carmine by 97% in the presence of acetosyringone within 2 h. Significantly increased activities of spore laccase, intracellular tyrosinase, and lignin peroxidase upon exposure to indigo carmine were observed. The results of RT–qPCR also showed that the expression of laccase gene was significantly increased. The spore has the ability to degrade indigo carmine through oxidization. Furthermore, the pathway by which indigo carmine is degraded was investigated using liquid chromatography–mass spectrometry analysis to identify the biodegradation products. A detailed pathway of indigo carmine degradation by bacterial spores was proposed for the first time. Toxicity tests indicated that the biodegradation products of indigo carmine are non-toxic to *Nicotiana tabacum* seeds and are less hazardous to human erythrocytes than the original dye. Indigo carmine is a typical recalcitrant dye and severely jeopardizes human health. The results demonstrate the utility of the spore from *Bacillus safensis* HL3 for the degradation of indigo carmine and simultaneous reduction of its toxicity.

## 1. Introduction

Dyes are widely employed in the textile, paper, leather, and pharmaceutical industries [1]. However, many dyes are known to be toxic and/or mutagenic to living organisms [2], and dye-colored industrial effluents present certain health hazards and environmental problems [3]. 

Indigo carmine (5,5′-indigodisulfonic acid sodium salt) is a typical dark-blue anionic isatin dye used to dye denim [4]. However, it is a recalcitrant dye and is severely damaging to organism health such as the injuring hepatocyte membranes [5], elevating blood pressure [6], etc. Therefore, the effective treatment of wastewater containing indigo carmine before discharge into the environment is mandatory [7].

Several processes have been employed to treat dye effluents, such as photolysis [8,9], electrooxidation [10], and the electro-Fenton process [11]. However, while these physical and chemical methods effectively degrade dyes, they generate secondary pollutants. Conversely, biological treatment methods have been shown to be both effective and environmentally friendly [2]. Such biological methods typically rely on naturally derived enzymes such as laccase to remove dyes from wastewater by degradation [12]. Biocatalysts have certain advantages over conventional catalysis owing to their high activities in aqueous solvents at ambient temperatures and pressures. In addition, they are renewable and nontoxic [13].

Laccases (benzenediol: oxygen oxidoreductases) catalyze one-electron oxidation of various substrates, concomitantly reducing molecular oxygen to water [14]. They are widely used in the biobleaching of kraft pulps, fiber modification in the paper industry, and removing dyes [15,16]. Bacterial spores are some of the most significant sources of laccase [17,18]. Spore laccase is a component of spore coat proteins located on the surface of the spore that can tolerate harsh environmental conditions. The spore made using simple treatment of bacterial cells can be regarded as an immobilized laccase and can be directly used as a biocatalyst without the addition of other components and may be removed simply from reaction mixtures [19,20]. Therefore, spore laccase is a highly economical biocatalyst. 

Bacterial spores have been previously applied to the degradation of dyes [21,22]. However, the degradation of indigo dyes remains poorly understood. Our group has studied indigo carmine, a representative indigo dye. Furthermore, Asadi et al. have utilized the spore from marine *Bacillus* sp. KC2 to study sporulation conditions, the characteristics of spore laccase, and the decolorization effect of a laccase mediator system on indigo carmine [20]. Nevertheless, the degradation pathway and the unambiguous identification and toxicity determination of the products of indigo carmine degradation by spore laccase have not been reported. 

Accordingly, in the current study, we investigated the feasibility of applying the spore from *Bacillus safensis* HL3 to the degradation of indigo carmine. Furthermore, the degradation products were determined by UV–Vis spectroscopy and liquid chromatography–mass spectrometry (LC–HRMS). Based on the degradation products identified, a decomposition pathway for indigo carmine by bacterial spore is proposed for the first time. Changes in the activities of various oxidases during the degradation of indigo carmine are also determined as a means to elucidate the degradation mechanism.

## 2. Results 

### 2.1. Isolation, Identification, and Biological Characteristics of the Strain 

Cu^2+^ was used as the reagent to screen the strains having laccase activity [23]. One colony from ten isolated strains, named strain HL3, was selected for further study because of its crimson-generating reaction with SGZ, which is a specific substrate of laccase. The strain HL3 is Gram-positive, and SEM observation showed that it is spore-forming and rod-shaped (0.44–0.48 μm × 1.07–1.67 μm) (Figure S1). 

**Figure 1:**
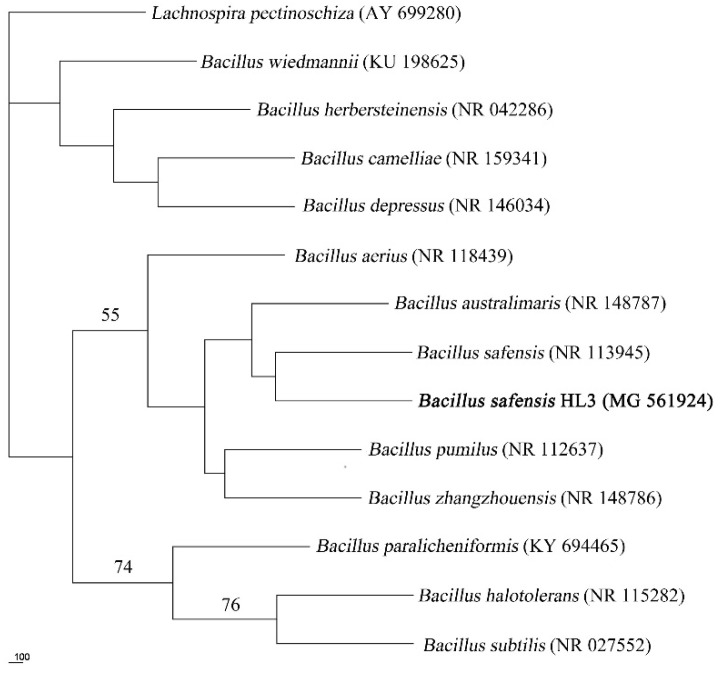
Phylogenetic tree for *Bacillus safensis* HL3 and related organisms were aligned based on 16S rDNA sequences (DNAPARS tree). Scale bar: number of nucleotide changes per sequence position. The number at nodes shows the bootstrap values obtained with 1000 resampling analyses.

The 1512-bp 16S rRNA gene of strain HL3 is available under the NCBI with accession number MG561924. The results of BLAST homology analysis showed that the strain has 99% homology with the genus *Bacillus*. In the phylogenetic tree (Figure 1), strain HL3 is closely related to *B. safensis*. The similarity of the 16S rDNA sequence supports the identification as *Bacillus safensis* HL3.

Strain HL3 is characterized by the following aspects: the optimum growth temperature and pH are 37 °C and 7.0, respectively (Figure S2a,b). It grows normally in 1%–10% NaCl (Figure S2c), and it has strong copper resistance, as it grows well in 0.2–0.8 mmol L^−1^ Cu^2+^ (Figure S2d). Strain HL3 can utilize monosaccharide glucose, fructose, mannose, mannitol, arabinose, and disaccharide sucrose. The results of physiological and biochemical tests are shown in Table S3.

### 2.2. Characterization of Spore Laccase

The activity of spore laccase from strain HL3 was found to be 35.66 U g^−1^ dry weight with SGZ as the substrate. The *Km* for SGZ, ABTS, and 2,6-DMP towards the spore laccase of strain HL3 are 0.035, 0.562, and 1.975 mmol L^−1^, respectively. Therefore, the affinities of the three substrates for spore laccase decrease in the order SGZ > ABTS > 2,6-DMP. The Vmax for ABTS, 2,6-DMP, and SGZ are 0.037, 0.031, and 0.014 mmol L^−1^ min^−1^, respectively. The optimum reaction temperature and pH of spore laccase are 80 °C and 7.0, respectively. Furthermore, spore laccase maintains 50% of its activity over the pH range 6.0–8.0. More than 70% activity is maintained at temperatures from 60–95 °C (Figure S3). 

The effects of metal ions, inhibitor, and organic reagents on the activity of spore laccase from strain HL3 were also determined. The laccase activity was increased in the presence of 10 mmol/L NH_4_^+^, K^+^, Ba^2+^, and Ca^2+^ and decreased in presence of the other metal cations, such as Fe^2+^, Mn^2+^, and Al^3+^ etc. (Table S4). Lower concentration of L-cysteine, EDTA, and SDS slightly improve the activity of spore laccase. Organic solvents inhibit spore laccases activity to a certain extent (Table S5). 

### 2.3. Indigo Carmine Degradation 

**Figure 2:**
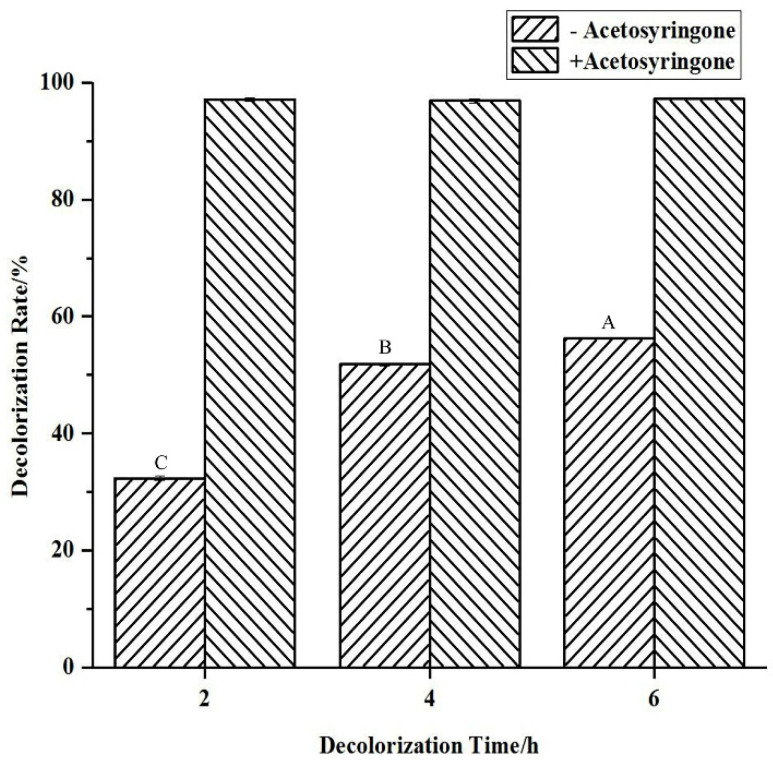
Decolorization rate of dye indigo carmine by the spore laccase of strain HL3 in the absence and presence of acetosyringone. Data were analyzed by one way ANOVA with a Tukey HSD multiple comparisons test using the means of three experiments. The different capital letters indicate significant differences at *p* < 0.01.

**Figure 3:**
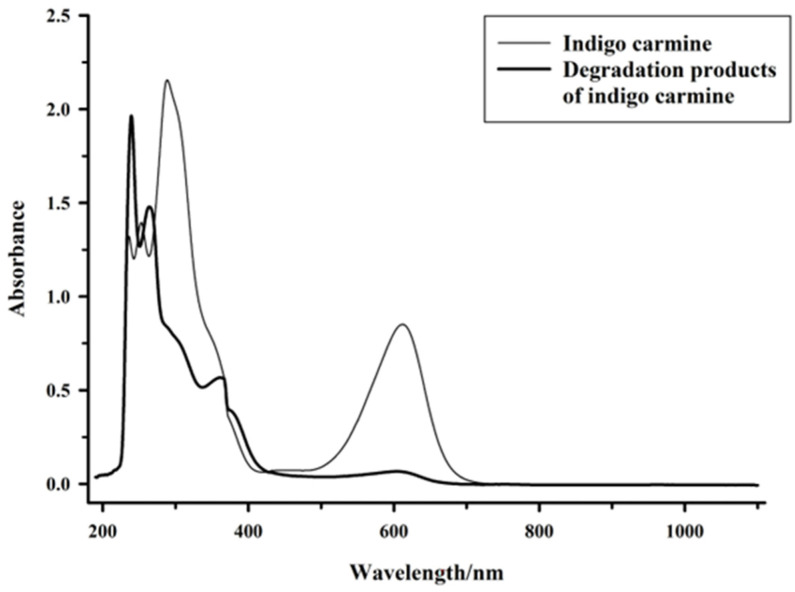
UV–Vis absorption spectra of indigo carmine before and after treatment by spore laccase.

The effects of the mediator acetosyringone on the decolorization rate were determined, and the changes in absorbance wavelength and LC–HRMS profile of the supernatant before and after decolorization were measured. Without the mediator, the decolorization of indigo carmine increases with time. However, in the presence of the mediator acetosyringone, the decolorization rate of indigo carmine greatly increases from 32% to 97% in 2 h (Figure 2). As shown in Figure 3, the UV–Vis spectrum (190–1100 nm) of the indigo carmine sample features peaks at 236, 253, and 287 nm in the UV region and at 610 nm in the visible-light region. In that of the indigo-carmine-treatment sample, the peak at 287 nm is no longer observed, while new UV bands (239, 262, and 360 nm) are observed.

The HPLC profile of the indigo carmine sample shows main peaks at 5.13 and 2.6 min (Figure S4a). However, the treated indigo carmine sample presents two new major peaks (retention times of 1.35 and 1.52 min) and several other detectable peaks (Figure S4b). The difference in the profiles of the indigo carmine sample and the treated sample indicates the transformation of the parent dye compound into new products.

**Figure 4:**
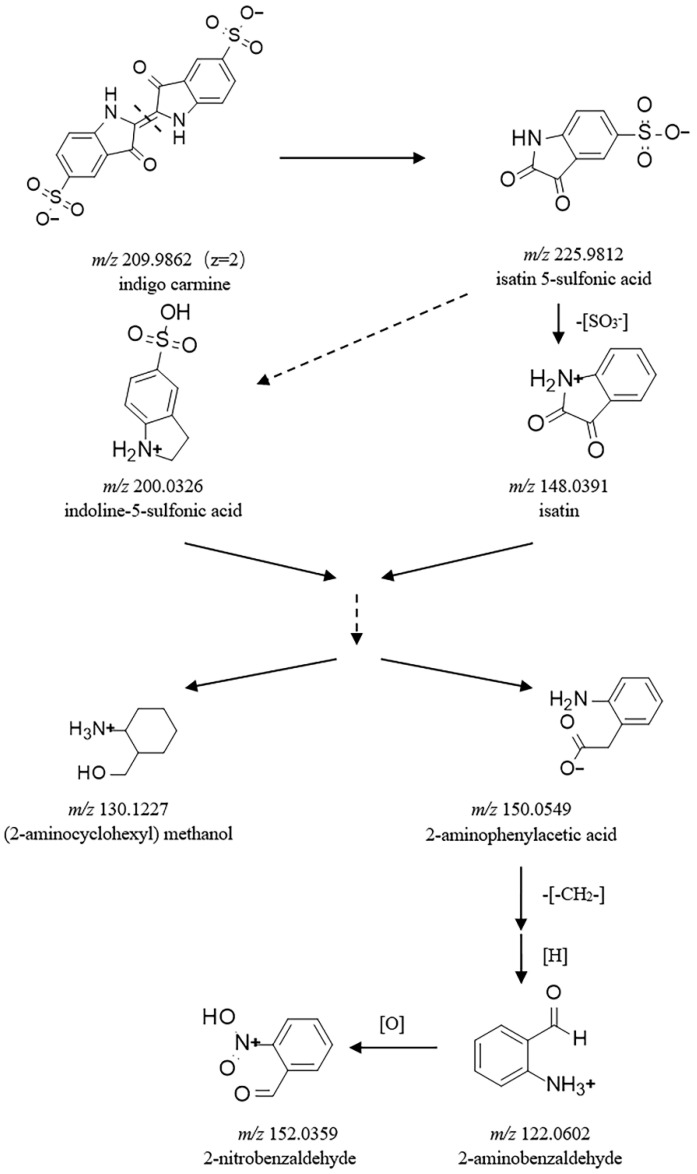
Proposed pathway for the biodegradation of indigo carmine using spore laccase of strain HL3.

The mass spectrometry and structural identification of indigo carmine sample and its biodegradation products are provided in Table 1 and Table S6. Using these data, we have proposed a pathway for the degradation of indigo carmine using the spore from strain HL3 (Figure 4). There is isatin 5-sulfonic acid (5-ISA, *m*/*z* 225.9812) in the indigo carmine sample, which indicates that the C=C double bond of indigo carmine is easy to break to form the intermediate product 5-ISA. In the degradation system, 5-ISA as the intermediate metabolite is subsequently degraded by the spore to aliphatic byproducts or alcohol compounds. Isatin (1H-indole-2, 3-diones, *m*/*z* 148.0391) is formed by desulfonation of 5-ISA [7,24]. The aromatic compound 2-nitrobenzaldehyde (*m*/*z* 152.0359) is formed by demethylation and redox from 2-aminophenylacetic acid (*m*/*z* 150.0549) [11,25,26] This is the first time the details of the degradation pathway of indigo carmine by spore laccase has been reported.

### 2.4. Analysis of Oxidase Activity during Biodegradation and RT-qPCR Validation

**Figure 5:**
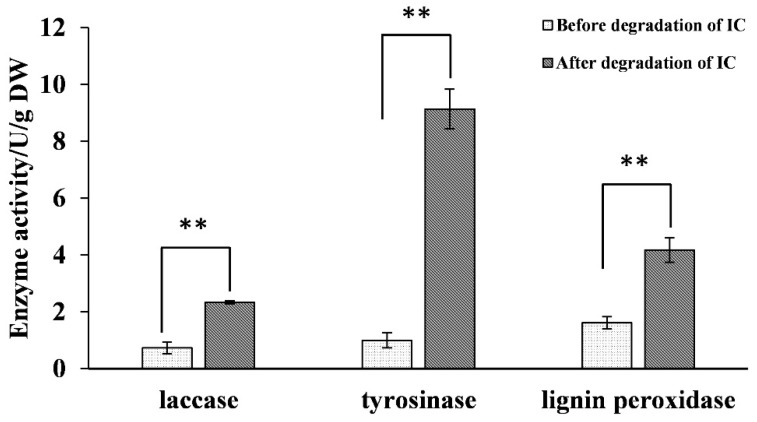
The activities of the oxidases from the spore of strain HL3 before and after indigo carmine treatment. Data were analyzed by paired *t*-test using mean values of three experiments. Two asterisks indicate significant difference at *p* < 0.01.

The changes in the activities of key oxidases in the supernatant of the degradation system, the cell-free extract, and the surfaces of spores were determined before and after indigo carmine degradation. The spore laccase activity from strain HL3 after the treatment of indigo carmine was higher significantly than that from control strain (at *p* < 0.01). Significantly increased intracellular tyrosinase activity and lignin peroxidase activity were also observed (at *p* < 0.01) (Figure 5).

To support the spore laccase activities results, RT–qPCR analysis of laccase genes from strain HL3 before and after the degradation of IC. At the end of PCR reaction, a dissolution curve analysis with the temperature decreased from 95 °C to 60 °C was carried out, and the change in the fluorescence signal in this process was monitored. The speed of the change in the fluorescence signal was plotted against the temperature to form dissolution curve. There was only one characteristic peak for 16S rDNA and laccase gene, respectively, and the *Tm* of the characteristic peak formed among 80–90 °C by the same gene was the same (Figure S5a,b). These indicated that no non-specific amplification or primer dimer was formed. Since the dye TB Green method is not specific for the recognition of double-stranded DNA, it is necessary to design the primers properly during the test. Therefore, six pairs of primers were designed by searching conserved sequence of laccase gene in *Bacillus*, and only primer pair no. 2 had specific amplification result. The amplification curve showed that the *Ct* of the laccase gene from strain HL3 after degradation of IC was lower than that of the control strain HL3 (Figure S5c). Compared with the control strain HL3, the laccase gene expression in the treatment strain HL3 was significantly increased by *t*-test analysis of paired samples (Table 2). These results supported the increasing laccase activities after the strain HL3 treated the dye IC.

### 2.5. Toxicity Analysis

**Figure 6:**
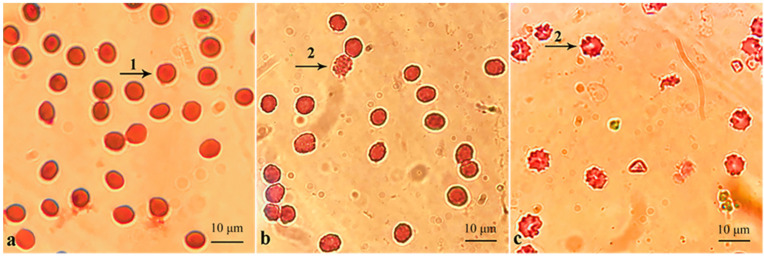
Photomicrographs of human erythrocytes from the control group (**a**), the group treated with the degradation products of indigo carmine (**b**), and indigo carmine alone (**c**). Arrow (1) shows smooth biconcave discoid erythrocytes and arrow (2) shows deformed erythrocytes.

The effects of indigo carmine and its degradation products on human erythrocytes and the germination rate of seeds were determined. The human erythrocytes in the control sample show smooth biconcave discoid shapes (Figure 6a). There are deformed erythrocytes (called echinocytes) in the treated samples (Figure 6b,6c). The echinocytes are fragile and more pinned compared with the normal erythrocytes. Echinocytes indicate damage to membrane proteins [27]. The numbers of normal erythrocytes after treating indigo carmine and the concentrations of indigo-carmine degradation products are decreased significantly compared with those of the control sample (*p* < 0.01) (Table 3). Indigo carmine inhibits *N. tabacum* seed germination more than the control (*p* < 0.05), while the degradation products of indigo carmine have a similar effect on the germination of *N. tabacum* to that of the control (Table 3).

## 3. Discussion

Usually, textile effluents exhibit alkaline pH and temperatures ranging from 35 to 60 °C [28,29]. Thus, the identification of a mesophilic strain having alkalophilic properties is a crucial step for efficient textile effluent treatment. However, most of the organisms used in dye wastewater treatment do not exhibit these properties. Bacterial consortium NBNJ6 exhibits maximum decolorization to direct Red 81 at a pH value around 7 [30]. However, *Bacillus safensis* HL3 grows well at 30–50 °C and pH 5–9. Salts such as NaCl are usually added to dye baths to increase ionic strength and dye fixation to fabrics. Thus, salts are also released along with the release of dye contaminants in textile wastewater [31]. Hence, the strain HL3 is suitable for treating industrial wastewater owing to its high tolerance to NaCl. Cu^2+^ is toxic to microorganisms and may cause death within minutes upon exposure [32]. For instance, *Bacillus* sp. SS4 is completely killed in 1 mmol L^−1^ Cu^2+^ [33]. However, *Bacillus safensis* HL3 survives under such conditions (Figure S2).

In industry, pretreatment of biomass before enzymatic treatment is performed in organic solvents with high purity and easy recovery [34]. Here, there is no need for the excessive washing of treated biomass with water before treatment with spore laccase as it is resistant to organic solvents [33]. These properties of the spore laccase of strain HL3 (Table S5) may allow it to be adapted for the bioremediation of harsh industrial wastewater at high temperature that contains ions and organic solvents [33,35].

In our study, the activity of spore laccase from strain HL3 was found to be 35.66 U g^−1^ dry weight with SGZ as the substrate, while the activity of that from *B. vallismortis* was 6.5 U g^−1^ dry weight with ABTS as the substrate [21]. According to Agrawal and Verma [36], the activity of laccase with ABTS as the substrate is 12 times that with SGZ. Thus, the activity of spore laccase from strain HL3 is much higher than that from *B. vallismortis*. It is more reliable in terms of the dry weight of spores to compare the activities of laccases from different strains. Mediators are relatively stable, low-molecular-weight compounds that expand the oxidative potential of laccases in the degradation of non-phenolic compounds by acting as carriers of electrons between laccase and the substrate [37]. In the presence of the mediator acetosyringone, the decolorization rate of indigo carmine greatly increases from 32% to 97% in 2 h (Figure 2). Acetosyringone as the mediator also improves the decolorization rate of indigo carmine by the spore laccase from *Bacillus* sp. KC2 [20].

The basic color-producing structure is a cross-conjugated system or H-chromophore [25]. The lack of the characteristic peak of the dye color at 610 nm for the supernatant taken from the decolorization system (Figure 3) is explained by the biocatalytic destruction of the characteristic chromophore group [24,38]. The decrease in peak intensity at 287 nm indicates the breakdown of the unsaturated double bonds present in the dye indigo carmine [38], while the appearance of the new UV bands (239, 262, and 360 nm) indicates that the cleavage of the dye molecule leads to an increase in aromatic groups [24]. According to Jadhav et al., the decolorization of indigo carmine by the spore from strain HL3 could be due to biodegradation, as evidenced by the fact that the major peaks disappear completely and new peaks appear [39].

Terminal desulfonation from indigo carmine is considered to be faster than the internal conversion [40]. Some treatment methods for the degradation of indigo carmine, such as photocatalytic degradation [41], photodegradation [42], O_3_, non-thermal plasma [7], bicarbonate-activated peroxide [43], Fenton-like [41], and fungal laccase [44], do not desulfonate indigo carmine. Nevertheless, complex or combined methods, such as hydroxyl radicals [26], Mg/ZnO-GO photocatalyst [45], electro-generated Cl_2_-active [46], gamma-radiation [24], and electro-Fenton [11] can degrade indigo carmine to form desulfonate byproducts. However, in our study, only the spore from strain HL3 was found to degrade indigo carmine by desulfonation (Figure 4). This indicates that the spore from strain HL3 has a strong oxidation capacity against indigo carmine.

Similar increases in activity in intracellular tyrosinase and lignin peroxidase have been reported for the biodegradation of Remazol Yellow RR [47], Reactive Black 5 [48], Remazol Orange 3R [49], Green HE4B [50], Brown 3REL [51], and Direct Red 5B [39]. Singh *et al*. speculated that indigo carmine increases laccase activity in γ-proteobacterium JB by increasing the level of its gene expression [52]. Increased levels in these oxidase activities affirmed the role played by enzymes during the degradation of these dyes. However, in the present study of indigo carmine degradation, we deduced that the spore laccase on the surface of strain HL3 plays the dominant role because intracellular tyrosinase and lignin peroxidase do not permeate out from the cell completely, though they are induced [39].

These results show that the erythrocyte membrane is very sensitive to indigo carmine and its degradation products, perhaps through the induced oxidation of lipids and proteins and enhanced osmotic fragility [53]. However, the degradation products are less hazardous to erythrocytes than the dye indigo carmine, because there are more normal erythrocytes in the post-degradation mixture that in the dye sample (*p* < 0.01). Younes et al. used the filtrate of bacteria to treat dyes, while in this study, the spores of bacteria were used [54]. Younes et al. showed that the filtrate of two bacteria reduced the toxicity of dyes to red blood cells, which was consistent with the results of this study.

Thus, it was deduced that the spore from strain HL3 degrades the toxic indigo carmine into non-phytotoxic products. Similar results have been reported for the biodegradation of Methyl Red [55], Brown 3 REL [51], and indigo carmine [54].

## 4. Materials and Methods

### 4.1. General

Premix Taq, *E. coli* DH5α competent cells, and pMD18-T vector were purchased from TaKaRa (Dalian, China); 2,2’-Azinobis-(3-ethylbenzthiazoline-6-sulphonate) (ABTS), syringaldazine (SGZ), 2,6-dimethoxyphenol (2,6-DMP), acetosyringone, and indigo carmine were purchased from Sigma-Aldrich (Shanghai, China). Bacterial microscale biochemical reaction tubes were obtained from Hangzhou Binhe Microbial Reagent Co., Ltd. (Zhejiang, China). Wright’s stain was obtained from Coolaber Science & Technology Co., Ltd. (Beijing, China).

### 4.2. Isolation, Identification, and Biological Characteristics of the Strain

The sample was collected from the soil around *Salix matsudana* in Gaolan County, Gansu Province. The strain was isolated from the soil sample using Cu^2+^ as an enrichment agent in M9 culture medium and screened by reaction with SGZ to evaluate laccase activity [56].

Morphological observation of the HL3 strain was made by scanning electron microscopy (SEM, FEI Quanta 200, Eindhoven, Netherlands). The physiological and biochemical characteristics were assessed using bacterial microscale biochemical reaction tubes. The 16S rRNA sequence analysis method [56] was used. Multiple sequence alignment was performed using Clustal X 2.1. A phylogenetic tree was constructed using DNAPARS in the Phylipwx package. The topology of the resultant tree was evaluated by bootstrap analysis with 1000 replicates.

The effects of temperature (25–50 °C), pH (3–10), NaCl (1–10%), and Cu^2+^ (0.2–4 mmol L^−1^) on the growth of the HL3 strain in LB medium were evaluated according to Wang et al. [56].

### 4.3. Characterization of Spore Laccase

The strain was cultured for sporulation in LB medium containing 0.2 mmol L^−1^ Cu^2+^ at 37 °C for 5–7 d. The spore suspension was prepared according to the procedure of Lu et al. [57]. The spores were isolated and purified by treated with 0.1 mg/mL lysozyme for 10 min at 37 °C, followed by ordinal washing with 1 mol L^−1^ NaCl, 0.14 mol L^−1^ NaCl, 0.1% (w/v) SDS, and deionized water, which all containing 10 mmol L^−1^ EDTA and 0.3 mg mL^−1^ phenylmethyl sulfonyl fluoride (PMSF). The spore suspensions were subjected to heat shocked at 80 °C for 10 min to remove thermal sensitive impurities.

Spores were formed after 5–7 d of culture. Individual vegetative cells not forming spores could be lysed by lysozyme cleavage the β-(1,4)-glycosidic bond between N-acetylmuramic acid and N-acetylglucosamine in the cell wall and osmotic cytolytic method of sodium chloride solutions. PMSF could inhibit protease activities to prevent inactivation of enzymes on the spore surface.

The spore laccase activity was determined in a 3 mL reaction system as follows: 500 μL of spore laccase suspension (equal to 0.00806 g dry weight) and 1 mmol L^−1^ SGZ as the substrate were mixed in 50 mmol L^−1^ disodium hydrogen phosphate–citrate buffer (pH 6.8). The corresponding system without spore laccase was used as the blank control. The methods used to define one unit of enzyme activity (U) were those of Wang et al. [58]. One mL of spore suspension was dried at 80 °C and the dry weight was used to calculate the activity of spore laccase per gram of dry spore.

Kinetic analysis of the spore laccase was carried out at 37 °C using ABTS (0.02–0.2 mmol L^−1^, ε_420_ = 3.6 × 10^4^ L mol^−1^ cm^−1^), 2,6-DMP (0.1–1 mmol L^−1^, ε_477_ = 4.96 × 10^4^ L mol^−1^ cm^−1^), or SGZ (1–10 mol L^−1^) as the substrate. The methods used to calculate *K_m_* were those of Taqieddin and Amiji [59]. The methods used to evaluate the effects of temperature, pH, inhibitor, relevant metal ions, and relevant organic reagents on the activity of spore laccase are those of Wang et al. [58].

### 4.4. Indigo Carmine Degradation

Decolorization of indigo carmine was explored in the presence and absence of acetosyringone as the mediator in 3 mL system. The final concentration of the mediator was 0.1 mmol L^−1^. The decolorization system containing a spore suspension of strain HL3 (equal to 3 mg dry weight), 47 mg L^−1^ indigo carmine, and disodium hydrogen phosphate -citrate buffer (pH 7.0) was incubated at 40 °C and 170 rpm for a predetermined period of time in the dark. The supernatant of the decolorization system was collected for absorbance measurement. The decolorization rate was determined by the decrease in absorbance at 608 nm and expressed in terms of percentage. To avoid interference from the mediator acetosyringone in the analysis of the degradation products, acetosyringone was not added to the indigo carmine degradation system. Thus, the treatment time was lengthened to 7 d. Control samples without spore were run in parallel.

To investigate the degradation products and pathway, the degradation mixture was centrifuged at 25 °C and 6000 rpm for 5 min to remove the spores. The supernatant was concentrated 10-fold in a rotary vacuum evaporator at 40 °C and 75 rpm. The supernatant was filtered and used for UV–Vis absorption and LC–HRMS analyses. UV–Vis absorption analysis was performed on a dual-beam UV–Vis spectrophotometer (N6000, Youke, Shanghai, China).

LC–HRMS analysis was performed on a Q Exactive Ultimate 3000 UPLC (Thermo Scientific, America) equipped with a Hypersil GOLD column (100 × 2.1 mm, 3 µm). The temperature of the chromatographic column was maintained at 30 °C. The mobile phases were (A) 0.1% formic acid/acetonitrile and (B) 0.1% aqueous formic acid. The gradient elution was performed as in Table S1 at 0.20 mL min^−1^. The injection volumes for the analysis of indigo carmine and its degradation products was 60 µL.

Electrospray ionization mass spectrometry (ESI+/-MS) analyses were performed in positive/negative ion mode. The ESI parameters were as follows: spray voltage: 3.2 kV; capillary temperature 300.00 °C; sheath gas flow: 40.00 Arb; aux gas flow: 15.00 Arb; spare gas: 0.00; max spray current: 100.00 µA; probe temperature: 350.00 °C; S-Lens RF level: 50.00%.

### 4.5. Oxidase Analysis during Indigo Carmine Degradation

#### 4.5.1. Preparation of Cell-Free Extract

The spores from strain HL3 were collected by centrifugation and resuspended in disodium hydrogen phosphate-citrate buffer (pH 6.8) for sonication (SCIENI-IID Ultrasonic cell comminution apparatus, Xinzhi Biotechnology Co., Ltd., Ningbo, China), keeping the sonicator output at 600 W, giving 300 strokes, each of 3 s with a 3 s interval. The supernatant collected by centrifugation was used as the enzyme source.

#### 4.5.2. Oxidase Detection

The activities of the oxidases from the cell-free extract and the supernatant of the decolorization system, as well as the oxidases on the surface of the spore, were assayed spectrophotometrically before and after indigo carmine treatment.

The activities of lignin peroxidase [60] and veratryl alcohol oxidase [61] were determined using veratryl alcohol as substrates. Oxidation of veratryl alcohol at 37 °C was monitored by an absorbance increase at 310 nm due to the formation of veratraldehyde (ε_310_ =9.3 × 10^3^ L mol^−1^ cm^−1^). Tyrosinase activity was determined using L-DOPA as a substrate [62]. Oxidation of L-DOPA at 37 °C was monitored by an absorbance increase at 475 nm due to the formation of dopaquinone (ε_475_ = 3.6 × 10^3^ L mol^−1^ cm^−1^). The activity of spore laccase was determined using SGZ. The enzyme activities were calculated by the dry weights of the spores.

### 4.6. RT-qPCR Validation

To verify the gene expression of the IC-induced spore laccase, RT-qPCR (real-time quantitative polymerase chain reaction) analysis of laccase gene was performed. Based on conservative mRNA sequences of laccase obtained from nucleotide databases in NCBI (National Center of Biotechnology Information), a total of six primer pairs targeting laccase genes were designed using SnapGene 4.3.6 software (San Diego, CA, USA). The gene encoding for 16s rDNA gene was used as an internal reference in all the reactions. The primers were designed based on a criterion that the resulting PCR products should be around 80–200 bp. These primers were synthesized by Shenggong Bioengineering Co., LTD, Shanghai, China, and the detailed information is provided in supplementary Table S2.

The primers were used to amplify laccase gene to check the specificity of the primers. After strain HL3 RNA extraction of control and treated, complementary DNA (cDNA) was prepared using total RNA with a HiFiScript cDNA Synthesis Kit (Conway Century Technology Co., LTD, Taizhou, China). Real-time quantification of mRNA was performed in triplicates on LightCycler 480 Ⅱ Real-Time PCR System (Roche Diagnostics GmbH, Germany) using TB Green^®^ Premix Ex Taq™ (Tli RNaseH Plus) kits (TaKaRa BioInc., Japan). The PCR was carried out in a 20-μL reaction mixture containing TB Green Premix Ex Taq (Tli RNaseH Plus), 70 ng of cDNA as the template, 4 pmol each of primer. The PCR condition was as follows: holding at 95 °C for 30 s, 40 cycles of denaturation (95 °C; 5 s), annealing and extension (60 °C; 30 s), followed by a melting curve at 95 °C for 5 s, 60 °C for 1 min, and finally at 55 °C for 30 s. 2^−ΔΔCt^ was employed to calculate the relative expression of laccase gene of strain HL3.

### 4.7. Toxicity Analysis

#### 4.7.1. Cytotoxicity to Human Erythrocytes

A human blood sample was collected from the fingertip of a healthy volunteer. The blood sample was washed with 0.01 mol L^−1^ PBS (pH 6.4–6.8) and centrifuged to remove the residual plasma. Then, 47 mg L^−1^ indigo carmine or the corresponding degradation products were added to the erythrocytes in PBS buffer (pH 6.4–6.8) and incubated for 60 min at 37 °C. A control was prepared without indigo carmine. Finally, the erythrocytes were spread onto slides [54]. The erythrocytes smears were air dried and stained using Wright’s stain. The slides were observed using an Olympus CX31 microscope, and the number of deformed erythrocytes were counted with a blood count board.

#### 4.7.2. Phytotoxicity to Seeds of Nicotiana Tabacum

Intact and healthy seeds of *Nicotiana tabacum* LJ0520 were selected, sterilized (30 s in 75% alcohol and 2 min in 9% NaClO solution), and washed with distilled water. Filtrates of indigo carmine and the corresponding degradation product were prepared. Ten mL filtrate was added to each petri dish, in which 50 seeds were placed evenly. A control was prepared with sterilized distilled water. Seeds were incubated at 26 °C and 70% relative humidity. When the radicle exceeded half the length of the seed, the seed was classified as germinated [54]. The seed germination rate (GR) was determined as the percentage of germinated seeds in the total number of seeds.

## 5. Conclusions

The spore from *Bacillus safensis* HL3 has the ability to degrade indigo carmine. The induced laccase on the surface of the spore of strain HL3 plays the dominant role in indigo carmine degradation, which proceeds by the terminal desulfonation and oxidization of indigo carmine to small molecule compounds. A detailed pathway of indigo carmine degradation by bacterial spores was proposed on the basis of the degradation products identified by LC–HRMS for the first time. The degradation products are nontoxic to tobacco seeds and hypotoxic to human erythrocytes. Hence, the spore from strain *Bacillus safensis* HL3 is a potential candidate for the degradation of indigo carmine contaminated water.

## Figures and Tables

**Table 1:** LC–HRMS data for indigo carmine and the products of its degradation by strain HL3.

Serial Number	Retention Time (min)	Adduct of the Substance	Experimental *m*/*z* of the Adduct	Theoretical *m*/*z* of the Adduct	Chemical Name
		Indigo carmine sample			
1	5.42	[M-2Na]^2−^	209.9862	209.9867	indigo carmine
2	5.32	[M-H]^−^	225.9812	225.9816	isatin 5-sulfonic acid
		Degradation products of indigo carmine			
1	17.56	[M+H]^+^	200.0326	200.0376	indoline-5-sulfonic acid
2	12.34	[M+H]^+^	148.0391	148.0393	isatin
3	11.68	[M+H]^+^	130.1227	130.1226	(2-aminocyclohexyl) methanol
4	9.35	[M-H]^−^	150.0549	150.0561	2-aminophenylacetic acid
5	9.63	[M+H]^+^	152.0359	152.0342	2-nitrobenzaldehyde
6	5.19	[M+H]^+^	122.0602	122.0600	2-aminobenzaldehyde

Note: All the standard deviation between experimental and theoretical *m*/*z* is less than 0.005.

**Table 2:** The results of RT-qPCR of laccase genes from strain HL3.

Sample	2^−ΔΔCt ††^
Control group	1.00 ± 0
Treatment group	2.60 ± 0.40 **

^††^ Values are a mean of three experiments ± standard deviation. Data are analyzed by *t*-test analysis of paired samples using means of three experiments. ** indicates significant difference at *p* < 0.01. Control group: laccase gene from strain HL3 before degradation of IC. Treatment group: laccase gene from strain HL3 after degradation of IC.

**Table 3:** Biotoxicity of dye indigo carmine (47 mg L^−1^) and its degradation products extracted after degradation (7 d).

	Normal Erythrocytes (×10^7^ mL^−1^) ^††^	Germination Rate of *N. tabacum* (%) ^††^
Control	5.15 ± 0.23 ^A^	53 ± 3 ^a^
IC	3.58 ± 0.08 ^C^	20 ± 5 ^b^
DP	4.43 ± 0.13 ^B^	52 ± 2 ^a^

^††^ Values are a mean of three experiments ± standard deviation. Data are analyzed by one way ANOVA with a Tukey HSD multiple comparisons test using means of three experiments. Means in each column with same letters are not significantly different. Different lowercase and capital letters indicate significant difference at *p* < 0.05 and 0.01, respectively. IC: indigo carmine, DP: degradation products of indigo carmine.

## Data Availability

The data presented in this work are available in the article and supplementary materials.

## Supplementary Materials

The following are available online at https://www.mdpi.com/article/10.3390/molecules27238539/s1, Figure S1: SEM image showing the morphological characteristics of strain HL3, Figure S2: Effects of temperature (a), pH (b), NaCl (c), and Cu2+ (d) on the growth of strain HL3, Figure S3: Effects of temperature (a) and pH (b) on the activity of spore laccase from strain HL3, Figure S4: (a) HPLC profile of the dye indigo carmine. (b) HPLC profile of the degradation products of indigo carmine, Figure S5: (a,b) The melting curves and (c) the amplification curves of RT–qPCR of laccase gene and 16S rDNA gene from strain HL3 before and after degradation of IC, Table S1: LC–HRMS gradient elution conditions, Table S2: The primer pairs targeting laccase and 16S rDNA genes, Table S3: Physiological and biochemical reactions of strain HL3, Table S4: Effects of metal ions on the activity of spore laccase from strain HL3, Table S5: Effects of inhibitors and organic reagents on the activity of spore laccase from strain HL3, Table S6: Mass peaks for indigo carmine and the products of its degradation by strain HL3.
