# Supplementary material for: The Biodegradation of Indigo Carmine by Bacillus safensis HL3 Spore and Toxicity Analysis of the Degradation Products
Source: Molecules. 2022 Dec 4;27(23):8539. doi: 10.3390/molecules27238539 (PMC9738463; doi:10.3390/molecules27238539)

Biodegradation of indigo carmine by *Bacillus safensis* HL3 spore and toxicity analysis of the degradation products

Chunlei Wang[1,2], Sijia Wang[1], Jieru Zhang[1], Shumin Jiang[1], Daizong Cui[1,2], Haiqiong Sun[3], Chengwei Liu[1,2], Lili Li[4], Min Zhao[1,2]*

[1] College of Life Sciences, Northeast Forestry University, Harbin 150040, China.

[2] Key Laboratory for Enzyme and Enzyme-like Material Engineering of Heilongjiang, Harbin 150040, China.

[3] Lanzhou Lansheng Plasma-derived Biotherapies Co., Ltd, Lanzhou 730000, China.

[4]Institute of Forestry Science of Heilongjiang Province, Harbin, 150040, China.

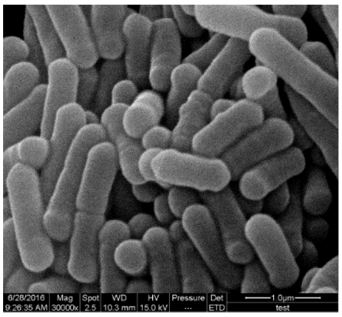

Figure S1 SEM image showing the morphological characteristics of strain HL3

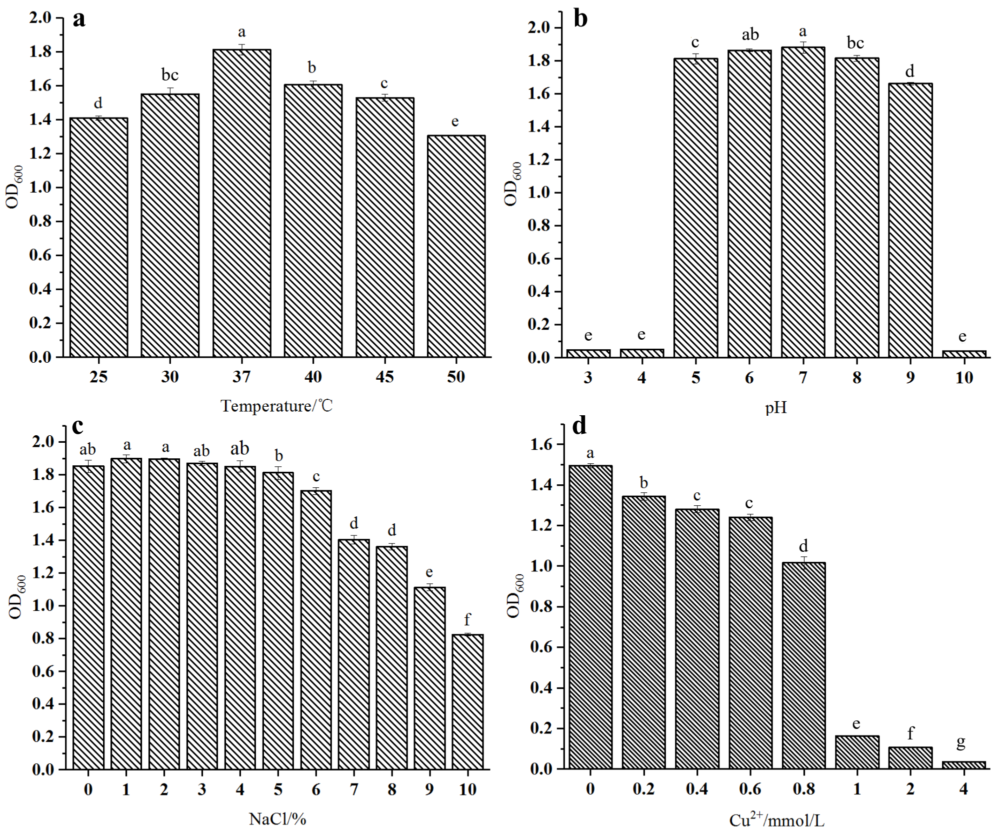

Figure S2 Effects of temperature (a), pH (b), NaCl (c), and Cu^{2+} (d) on the growth of strain HL3. Data were analyzed by one way ANOVA with a Tukey HSD multiple comparisons test using mean values of three experiments. The different lowercase letters indicate significant difference at $p < 0.05$.

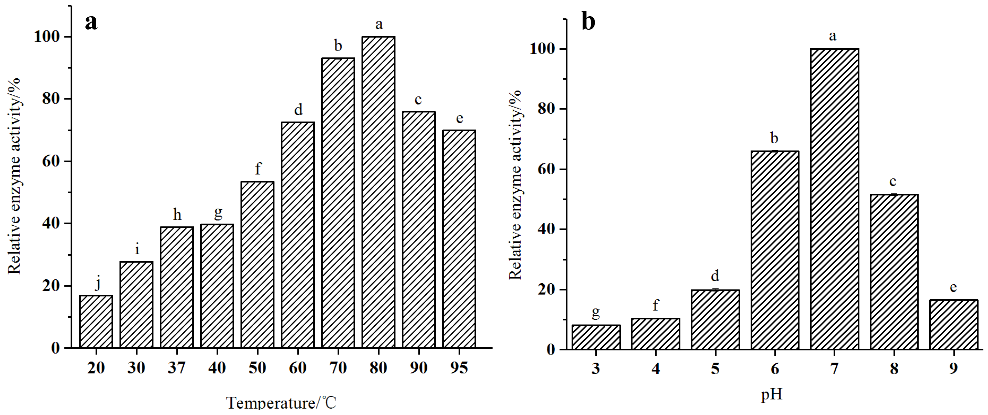

Figure S3 Effects of temperature (a) and pH (b) on the activity of spore laccase from strain HL3. Data were analyzed by one way ANOVA with a Tukey HSD multiple comparisons test using mean values of three experiments. The different lowercase letters indicate significant difference at $p < 0.05$.

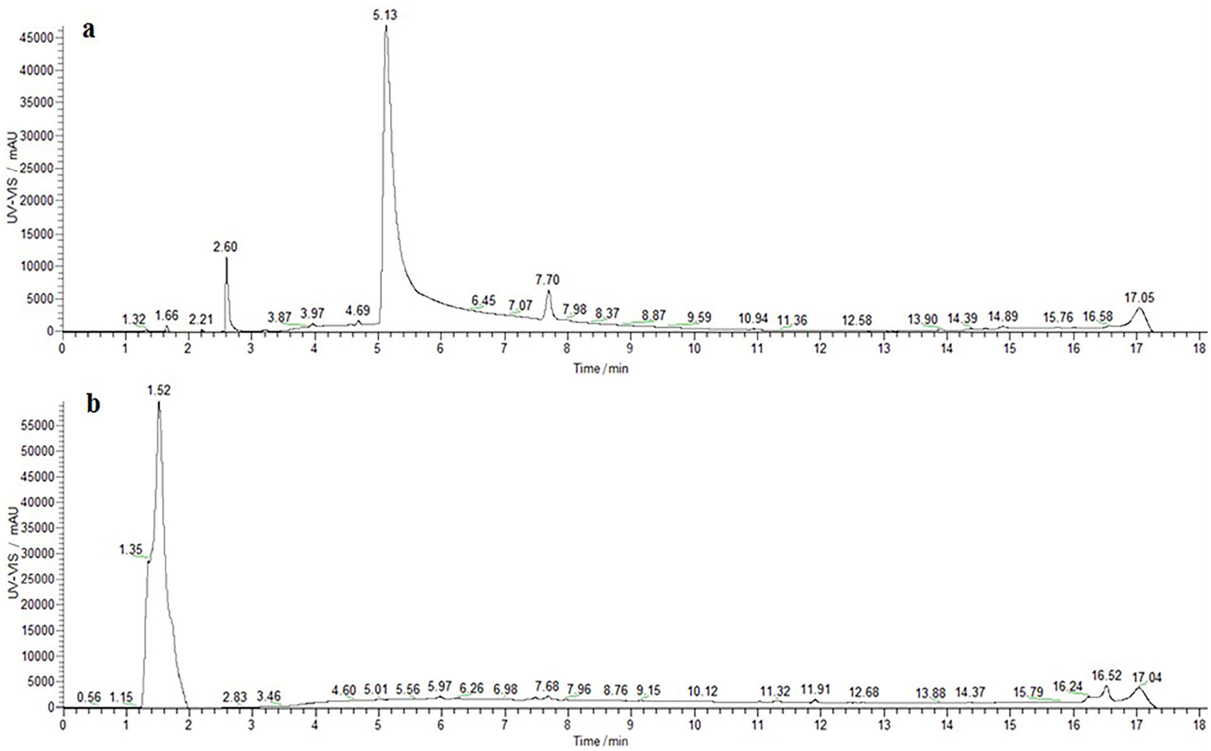

Figure S4 (a) HPLC profile of the dye indigo carmine. (b) HPLC profile of the degradation products of indigo carmine.

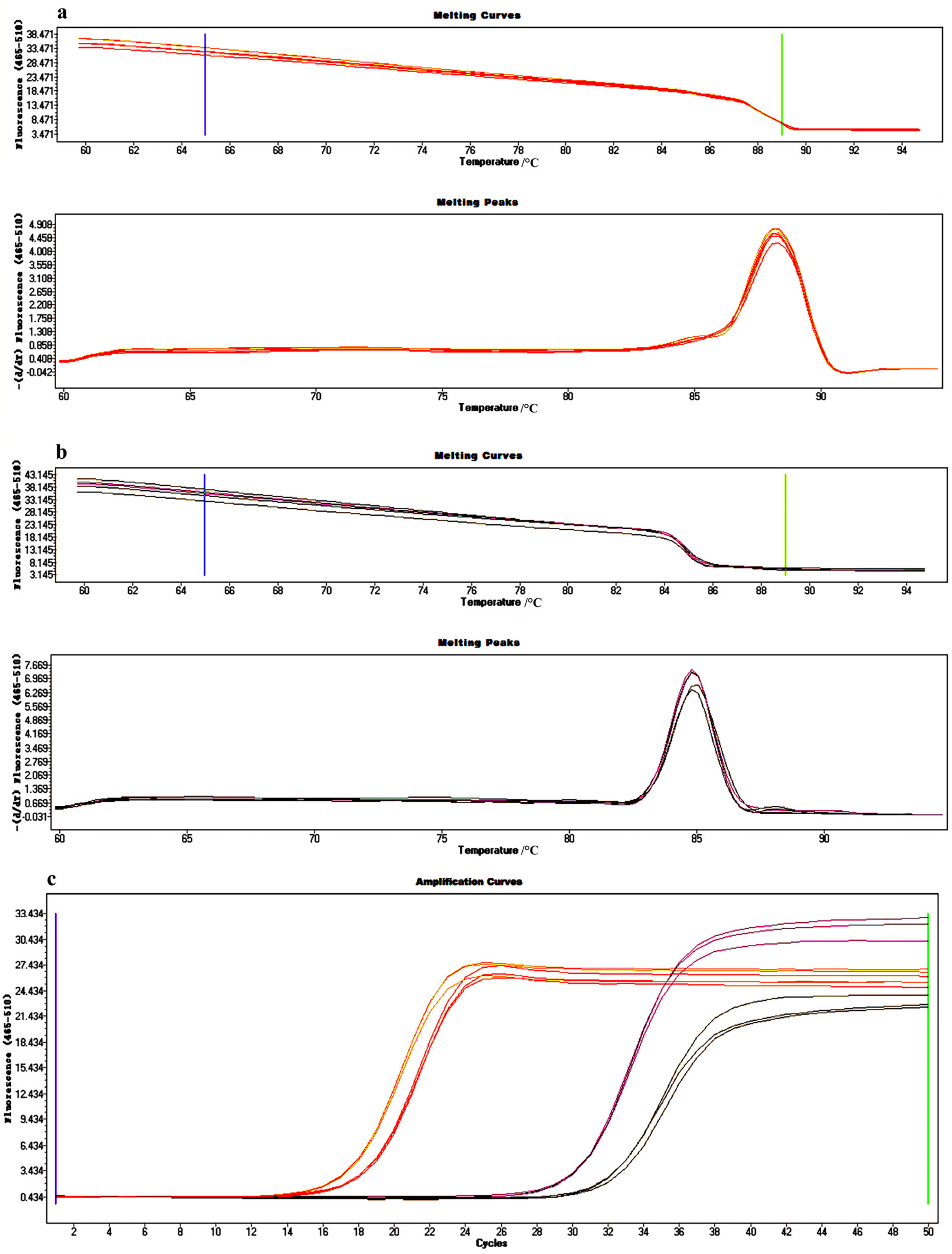

Figure S5 (a,b) The melting curves and (c) the amplification curves of RT–qPCR of laccase gene and 16S rDNA gene from strain HL3 before and after degradation of IC. Red: 16S rDNA before degradation of IC, Orange: 16S rDNA after degradation of IC, Black: laccase gene before degradation of IC, Purple: laccase gene after degradation of IC.

Table S1 LC–HRMS gradient elution conditions.

Time (min)	A (%)[a]	B (%)[b]
0	5	95
12	80	20
14	80	20
14.1	5	95
22	5	95

a 0.1% formic acid in acetonitrile.

b 0.1% aqueous formic acid.

Table S2 The primer pairs targeting laccase and 16S rDNA genes.

Primer	Upstream primer (5`-3`)	Downstream primer (5`-3`)
For *cotA*		
1	CGATGTCATCATCGACTTTTCAGCT	GGACGTGTGACTTTGAATTGCAT
2	GCACATTGTGGTATCACGATCAT	AACAGTGCGCCATCCTCCT
3	CAATTTGCTTGACGGTTCCT	GACCAAACACACGAGGAT
4	GTCCTTGTGACATCTCGGAAT	CAAGTCGTCATTGGACCATCCAT
5	GTATACGCCGGATTAGCTGGCT	CTGGAATGTCCGGTCCATGAT
6	CATTCACGCTGGACACCATGAT	GCATGCTTGCTGGTGATTAGGGT
For *16S rDNA*		
1	GAAGCTGGAATCGCTAGTAATC	TACGGCTACCTTGTTACGACTT

Table S3 Physiological and biochemical reactions of strain HL3.

Test item	Result	Test item	Result
Mannose	positive	Xylitol	negative
Fructose	positive	Sorbitol	negative
Glucose	positive	Raffinose	negative
Mannitol	positive	Sorbose	negative
Arabinose	positive	Simmons Citrate	negative
Sucrose	positive	H_2S production	negative
Inositol	negative	Arginine dihydrolase	positive
Rhamnose	negative	Starch hydrolase	negative
Galactose	negative	Gelatin hydrolase	positive
Melibiose	negative	O-F of glucose	fermentation
Maltose	negative	V-P test	positive
Xylose	negative	Nitrate reduction	negative
Lactose	negative	Nitrate aerogenesis	positive

Table S4 Effects of metal ions on the activity of spore laccase from strain HL3.

Metal ion	Concentration (mmol L^{-1})	Residual enzyme activity(%)[a]	Metal ion	Concentration (mmol L^{-1})	Residual enzyme activity(%)[a]
NH_4^+	10	104.57 ± 0.98	Ni^{2+}	10	53.55 ± 0.79
K^+	10	102.17 ± 0.93	Fe^{2+}	10	21.61 ± 0.47
Ba^{2+}	10	101.60 ± 0.97	Mn^{2+}	10	14.42 ± 0.36
Ca^{2+}	10	101.40 ± 1.05	Cu^{2+}	10	68.90 ± 0.50
Na^+	10	98.71 ± 1.17		20	62.07 ± 0.47
Mg^{2+}	10	92.32 ± 1.12		30	58.48 ± 0.83
Al^{3+}	10	77.13 ± 2.22		40	51.69 ± 0.92
Co^{2+}	10	73.30 ± 0.78		50	40.46 ± 0.36
Fe^{3+}	10	68.30 ± 0.82		60	8.09 ± 0.02
Zn^{2+}	10	67.20 ± 0.38			

a Values are a mean of three experiments ± standard deviation.

Table S5 Effects of inhibitors and organic reagents on the activity of spore laccase from strain HL3.

Reagents	Concentration (mmol L^{-1})	Residual enzyme activity(%)[a]	Reagents	Concentration (mmol L^{-1})	Residual enzyme activity(%)[a]
Inhibitors					
NaCl	200	90.31 ± 2.58	EDTA	1	112.10 ± 1.22
	500	70.70 ± 1.26		10	92.58 ± 1.11
	1,000	32.61 ± 2.09		50	57.31 ± 0.72
	1,500	8.53 ± 1.51		100	5.66 ± 0.21
	2,000	6.03 ± 0.10		200	5.39 ± 0.09
SDS	1	117.72 ± 0.90	L-cysteine	0.1	110.13± 1.57
	10	98.54 ± 1.30		1	9.89 ± 0.10
	50	75.00 ± 0.73		10	8.59 ± 0.09
	100	66.80 ± 0.38			
Organic reagents					
Acetone	10	90.05 ± 0.96	Ethanol	10	70.33 ± 0.69
	20	69.53 ± 0.60		20	44.29 ± 0.92
	30	39.56 ± 0.17		30	33.60 ± 0.52
Methanol	10	85.79 ± 0.79	Dimethyl sulfoxide	10	62.81 ± 0.49
	20	59.14 ± 0.55		20	43.99 ± 0.09
	30	47.69 ± 0.70		30	31.07 ± 0.36
Acetonitrile	10	76.23 ± 1.18			
	20	46.05 ± 0.18			
	30	42.66 ± 0.31			

a Values are a mean of three experiments ± standard deviation.

Table S6 Mass peaks for indigo carmine and the products of its degradation by strain HL3.

Serial number	Chemical name	Mass peaks (showing *m/z* value)
Indigo carmine sample		
1	indigo carmine	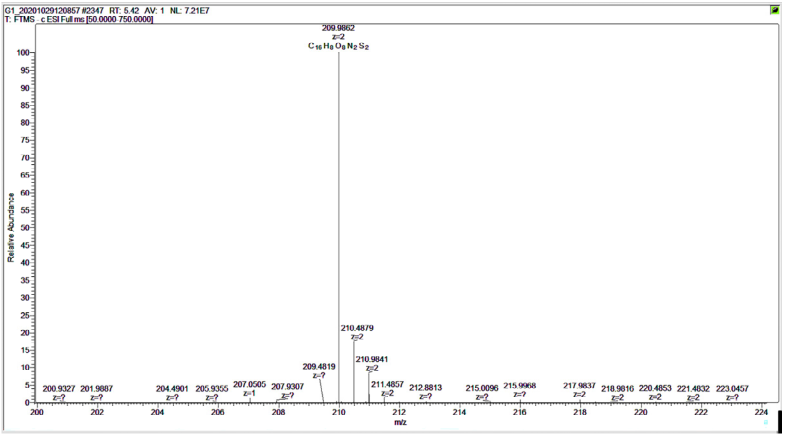
2	isatin 5-sulfonic acid	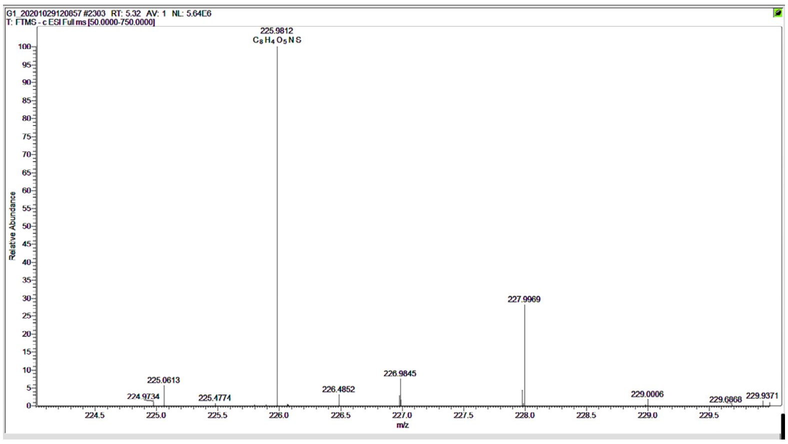
Degradation products of indigo carmine		

1 indoline-5-sulfonic acid

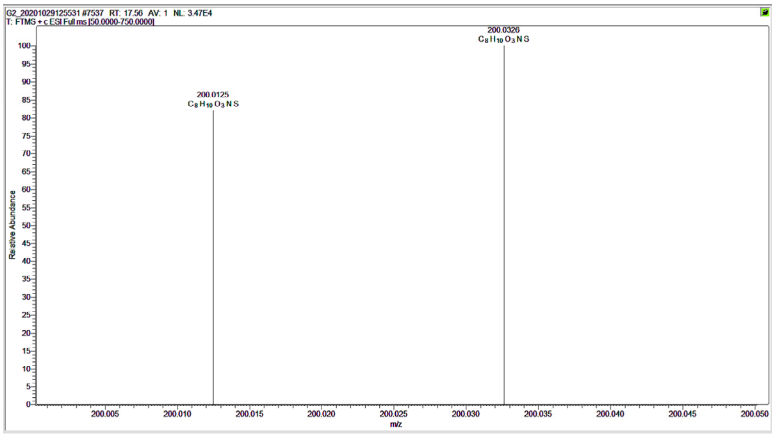

2 isatin

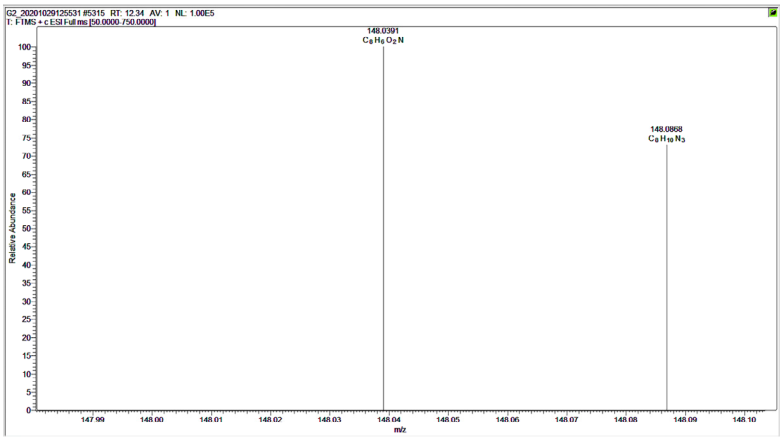

3 (2-aminocyclohexyl) methanol

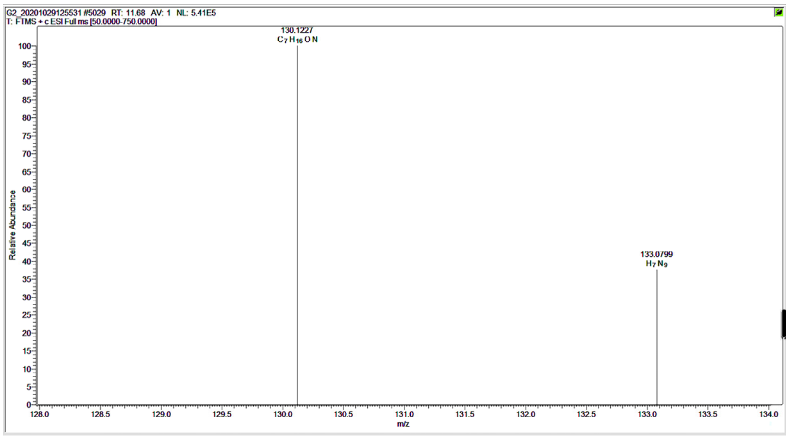

4 2-aminophenylacetic acid

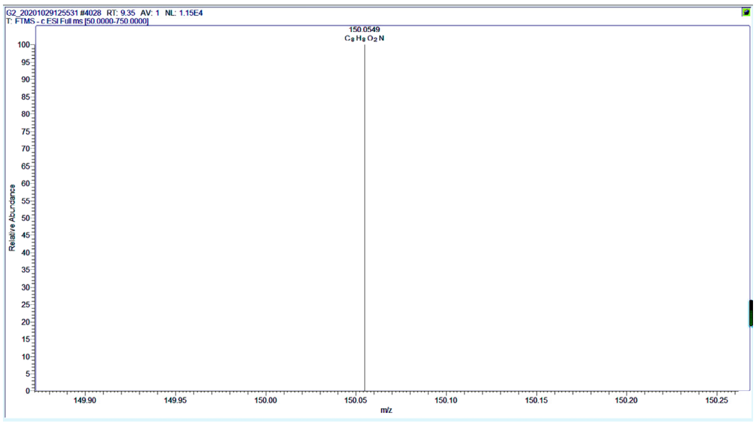

5 2-nitrobenzaldehyde

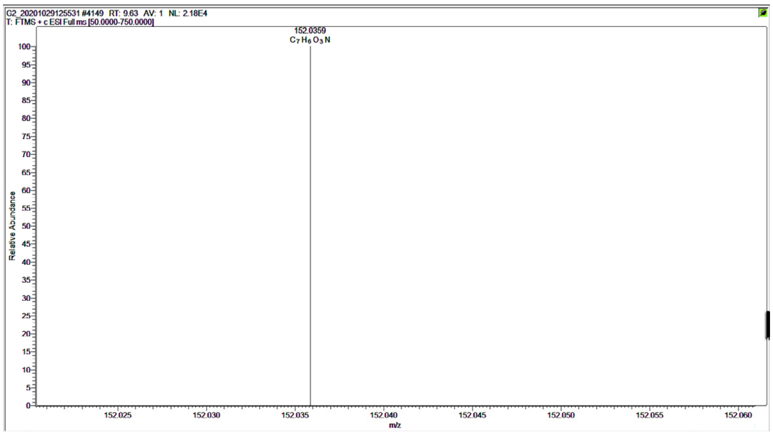

6 2-aminobenzaldehyde

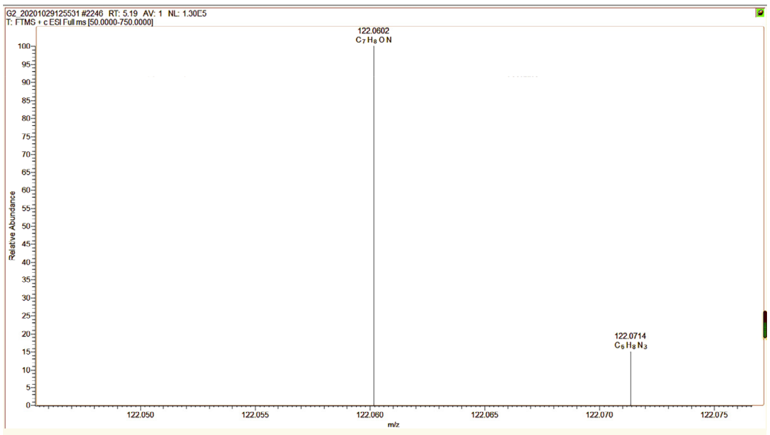

Supplement: Supplementary file 1 [file molecules-27-08539-s001.zip › molecules-2018219-supplementary.pdf]
